# Does metformin exposure before ICU stay have any impact on patients’ outcome? A retrospective cohort study of diabetic patients

**DOI:** 10.1186/s13613-017-0336-8

**Published:** 2017-12-02

**Authors:** Sebastien Jochmans, Jean-Emmanuel Alphonsine, Jonathan Chelly, Ly Van Phach Vong, Oumar Sy, Nathalie Rolin, Olivier Ellrodt, Mehran Monchi, Christophe Vinsonneau

**Affiliations:** 1 Département de Médecine Intensive et Unité de Recherche Clinique, Groupe Hospitalier Sud Ile-de-France, Hôpital de Melun, 77000 Melun, France; 2 Service de Réanimation Médicale, AP-HP, Hôpital Bicêtre, 94270 Le Kremlin-Bicêtre, France; 3 Département de Médecine Intensive, Groupe Hospitalier Sud Ile-de-France, Hôpital de Melun, 77000 Melun, France; 4 Service de Réanimation Polyvalente, Hôpital de Bethune, 62408 Bethune, France

**Keywords:** Metformin, Septic shock, Diabetes, Lactic acidosis, ICU

## Abstract

**Background:**

Impact of metformin exposure before ICU stay remains controversial. Metformin is thought to induce lactic acidosis and haemodynamic instability but may reduce ICU mortality. We evaluated its influence on outcome in diabetic patients admitted in the ICU and then compared two different populations based on the presence of septic shock.

**Methods:**

We conducted a retrospective cohort study in a 24-bed French ICU between October 2010 and December 2013, including all ICU-admitted diabetic patients.

**Results:**

Among 635 diabetic patients admitted during the study period, 131 (21%) were admitted with septic shock. Multivariate analysis showed no difference in hospital mortality in all metformin users (OR 0.75 [95% CI 0.44–1.28]; *p* = 0.29), except in the septic shock subgroup (OR 0.61; 95% CI [0.37–0.99]; *p* = 0.04) despite higher vasopressor dosages in the first hours after shock onset. Blood lactate level was higher in metformin users than in non-metformin users in all patients (*p* < 0.001), in septic shock patients (*p* < 0.001) and in patients without kidney injury (*p* < 0.001). Metformin users did not have more septic shock from unknown aetiology (*p* = 0.65) or unknown pathogen (*p* = 0.99).

**Conclusions:**

Metformin use before admission to ICU did not affect in-hospital mortality. However, for patients with septic shock, mortality was lower, despite worse clinical presentation on admission. Blood lactate levels were always higher with or without septic shock and indifferent of kidney function.

**Electronic supplementary material:**

The online version of this article (10.1186/s13613-017-0336-8) contains supplementary material, which is available to authorized users.

## Background

Metformin is increasingly used as an oral antidiabetic (OAD) agent, especially in patients with type 2 diabetes mellitus. Metformin inhibits hepatic glucose production, reduces intestinal glucose absorption and improves glucose metabolism [1].

Its use is associated with a reduction in cardiovascular morbidity and mortality, in comparison with insulin, other OADs or diet alone, in non-acutely ill patients [2, 3]. It is thought to induce or worsen lactic acidosis, especially in acute renal or liver dysfunction [4]. But in a recent meta-analysis pooling 347 trials involving long-run metformin use, the authors found no case of metformin-associated lactic acidosis (MALA), as well as no difference in blood lactate level related to metformin use [5]. These results were confirmed in a large cohort of diabetic patients treated with metformin despite various metformin contraindications, in which no MALA has been described by the authors [2].

In the ICU, MALA has been described in renal, liver, pulmonary or cardiovascular chronic failure [6], and several case reports described fatal or non-fatal MALA in acute conditions. In contrast, a recent retrospective study in 17 Danish ICUs found that prior to admission metformin use was associated with a reduction in 30-day mortality [3].

Our main objective in this study was to evaluate the influence of pre-admission metformin use on outcome in diabetic ICU patients and in a subgroup experiencing septic shock (an acute condition known to induce lactic acidosis [7, 8]). Secondary objectives were to assess MALA incidence and blood lactate levels in ICU patients with diabetes, treated or not by metformin, with or without septic shock.

## Methods

We performed a retrospective cohort study in our Intensive Care Medicine Department between October 2010 and December 2013. The study protocol was approved by the French Intensive Care Society (FICS)—Société de Réanimation de Langue Française (SRLF)—ethical review board.

### Patients

#### Inclusion and exclusion criteria

All patients admitted within the study period with a history of diabetes treated by insulin or oral antidiabetics were included. So-called diabetic patients treated only with diet were considered as unconfirmed diabetes and were excluded. The other exclusion criteria were as follows:

Unknown chronic antidiabetic treatment, modifications of antidiabetic treatment during the month before ICU admission and unavailable arterial blood gas sample within 4 h after ICU admission.

### Data collection

Collected clinical features were as follows: age, sex, height, weight, Simplified Acute Physiology Score II (SAPS II), main admission cause, metformin contraindication (‘Definitions’ paragraph below), ICU admission biomarkers (leucocytes, platelets, haemoglobin, creatinine, C-reactive protein, bilirubin and/or INR if available), arterial blood gas samples at day 1, all bacteriological tests, vasopressor dosages (close to the initiation even outside the ICU), urinary output and amount of intravascular input during the first 24 h, the use of invasive ventilation and renal replacement therapy, the presence of acute respiratory distress syndrome (ARDS), ICU and hospital length of stay and vital status.

### Definitions

Usual metformin contraindications (adapted from the instructions for the use of the medicinal product) were defined as:

1. Chronic respiratory failure (previous pulmonary function tests, history of acute respiratory decompensation, oxygen or non-invasive ventilation at home, sleep apnoea) and/or
2. Chronic cardiac failure (history of pulmonary oedema, left ventricular ejection fraction < 45%) and/or
3. Chronic renal disease (calculated creatinine clearance with Modification of Diet in Renal Disease [MDRD] < 60 mL/min/1.73 m^2^) and/or
4. Chronic liver disease (history of cirrhosis, previous INR > 1.2) and/or
5. Myocardial infarction during the previous month

Septic shock was defined according to the Surviving Sepsis Campaign definition [9]. Acute kidney injury was defined using Kidney Disease Improving Global Outcome (KDIGO) classification [10] and was considered for any stage of the classification.

### Statistical analysis

Continuous variables were expressed as median [25th–75th interquartile range] or mean ± standard deviation [95% confidence interval] (after Shapiro–Wilks test) and compared using nonparametric Mann–Whitney (or Student’s *t* test) and linear regression tests. Categorical variables were expressed as *n* (%) and compared using Chi-square or Fisher’s exact tests. All tests were two-tailed assuming alpha risk = 0.05. All collected data were analysed in univariate analysis regarding ICU and hospital survivals. We included in forward and backward stepwise multivariate regression models as covariates all data with *p* < 0.1 in univariate analysis, with stratification by metformin use. We applied these models in ICU patients and in the subgroups of septic shock and metformin users with usual contraindication. We performed a post hoc validity assessment of the regression models by receiver operating characteristic (ROC) curves, and we selected as the result the model with the best area under the curve. Results of multivariate regression test were expressed by odds ratio (95% confidence interval). Prognostic value of blood lactate level on mortality was tested with ROC curves (results expressed by area under the curve [AUC] % (95% confidence interval)), sensitivity and sensibility.

Statistical analysis and graphic representations were performed with SPSS Statistics V20 software (IBM^®^, New York, NY, USA) and Prism 6 software (GraphPad Software Inc.^®^, San Diego, CA, USA).

## Results

Among the 3871 patients admitted in our ICU during the study period, 635 (16.4%) were finally included (study flowchart is available in Additional file 1: Figure S1), including 131 (20.6%) patients with septic shock at day 1 after ICU admission.

Metformin use before admission was found in 240 patients (37.8%) and was similar regarding occurrence or non-occurrence of septic shock (*p* = 0.69). Ratio of metformin use in patients with one or more usual contraindications was high (119 (49.6%)) with a similar rate in septic shock patients (*p* = 0.54).

### ICU admission and hospital stay

The main characteristics of ICU diabetics at admission and during ICU or hospital stays are specified in Table 1 and Additional file 1: Table S1. In our study cohort, 588 (92.6%) patients were admitted for a medical cause, mainly for acute respiratory failure (266 (41.9%)). There was no difference between metformin users (MET) and non-metformin users (NO-MET) in the reason for admission. MET were younger with less chronic respiratory and renal failures. They had higher blood lactate level (*p* < 0.001), lower bicarbonate (*p* < 0.01) and also lower serum creatinine (*p* < 0.001) with less acute kidney injury (*p* < 0.001). Severity score (SAPS II) and need in organ support (i.e. invasive mechanical ventilation, vasopressor, renal replacement therapy) were similar. Among MET, there was no difference in lactate level between patients with or without usual contraindication (*p* = 0.86) (Additional file 1: Table S2).

**Table 1:** Cohort of ICU diabetics: main characteristics at ICU admission, during ICU stay and ICU/hospital outcome

	ICU diabetics	No metformin	Metformin
N	635	395 (62.2)	240 (37.8)
Age (y)	71 [61–79]	73 [62.5–80]	68 [60–78]*
Men	408 (64.3)	255 (64.6)	153 (63.8)
SAPS II	39 [31-52]	40 [32-52]	38 [29-51]
Usual metformin contraindication	387 (60.9)	268 (67.9)	119 (49.6)*
Chronic respiratory insufficiency	190 (29.9)	132 (33.4)	58 (24.2)*
Chronic cardiac insufficiency	138 (21.7)	92 (23.3)	46 (19.2)
Chronic liver disease	75 (11.8)	49 (12.4)	26 (10.8)
Chronic kidney failure	144 (22.7)	128 (32.4)	16 (6.7)*
Recent myocardial infarction	8 (1.3)	5 (1.3)	3 (1.3)
pH	7.36 [7.28–7.42]	7.36 [7.29–7.43]	7.36 [7.27–7.42]
PaCO_2_ (mmHg)	36 [29–43]	37 [30–44]	36 [28–43]
HCO_3_ (mmHg)	21.3 [17–25.2]	21.9 [17.5–26]	20.4 [15.3–24]*
Lactate (mmol/L)	1.4 [0.9–2.4]	1.2 [0.8–2.1]	1.8 [1.1–3.9]*
INR	1.25 [1.06–1.71]	1.26 [1.06–1.65]	1.24 [1.07–1.77]
Bilirubin (µmol/L)	10 [7–16]	10 [7–16]	10 [7–16]
C-reactive protein (mg/L)	34 [8–115]	35 [8–115]	32 [8–115]
Haemoglobin (g/dL)	11.2 [9.6–13]	11.1 [9.6–12.7]	11.7 [9.7–13.4]
Leucocytes (G/L)	11.2 [8.1–15.3]	10.8 [7.5–14.6]	11.7 [8.4–16.3]
Platelets (G/L)	213 [155–277]	207 [155–271]	219 [157–293]
Creatinine (µmol/L)	131 [85–238]	153 [90–285]	108 [80–174]*
Acute kidney injury	392 (61.7)	268 (67.8)	124 (51.7)*
Renal replacement therapy	113 (17.8)	72 (18.2)	41 (17.1)
Vasopressors	229 (36.1)	136 (34.4)	93 (38.8)
Invasive ventilation	230 (36.2)	139 (35.2)	91 (37.9)
ICU length of stay (d)	6 [3–10]	6 [3.5–10]	6 [3–9]
ICU death	117 (18.4)	75 (19)	42 (17.5)
Hospital length of stay (d)	12 [6–23]	12 [6–23]	13 [7–23]
Hospital death	140 (22)	92 (23.3)	48 (20)

Values are *n* (%) or median [IQR 25th–75th]

* *p* < 0.05 between metformin and no metformin

**Fig. 1:**
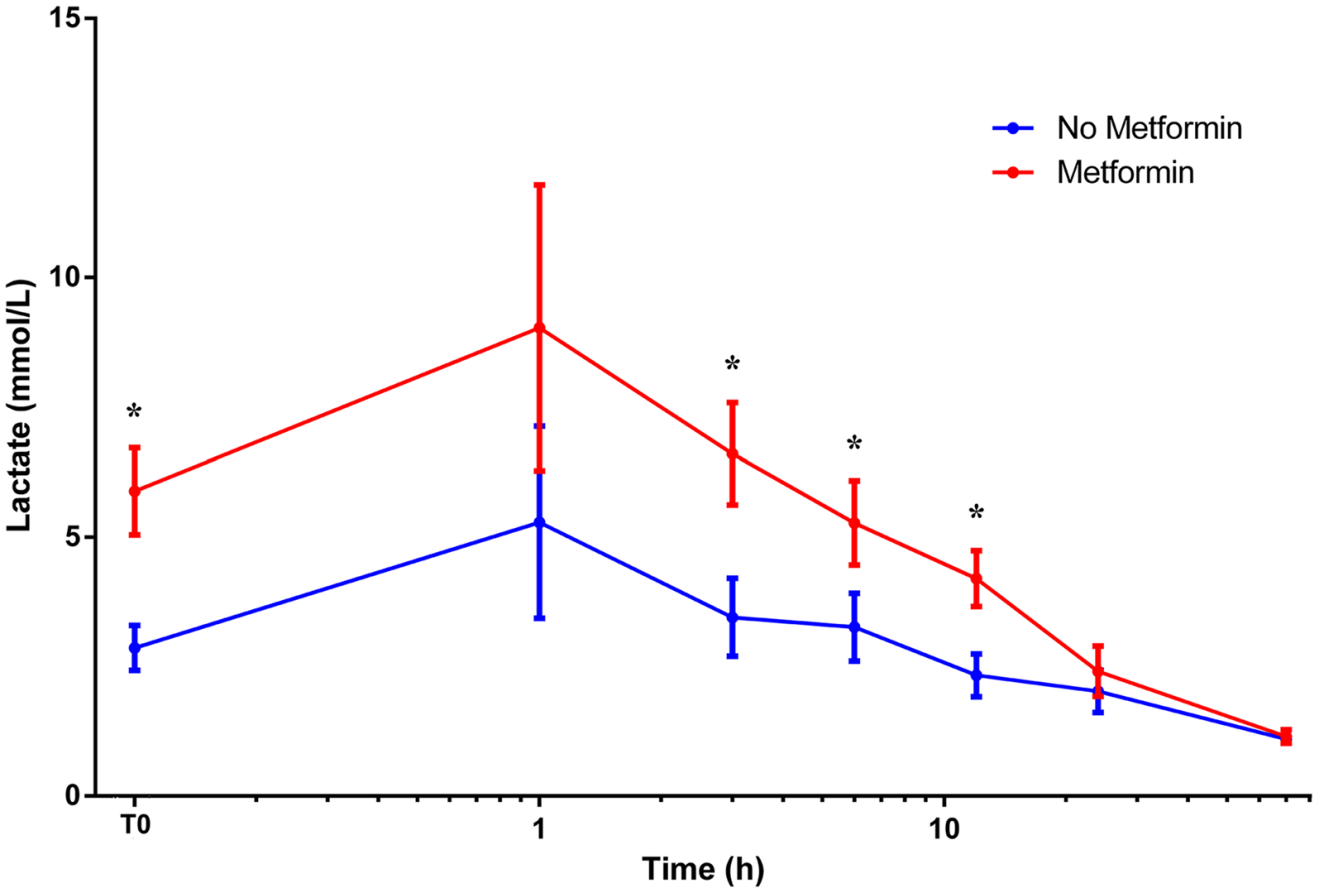
Initial evolution of lactate level in ICU diabetics sustaining septic shock with or without pre-admission metformin treatment. T0: time of septic shock diagnosis. Abscissa axis is log 10 scale. *p < 0.05

The main characteristics for diabetics with septic shock at admission and during ICU or hospital stays are specified in Table 2 and Additional file 1: Tables S3 and S4. Aetiologies of shock are specified in Additional file 1: Table S3. There was no difference between MET and NO-MET regarding unknown aetiology (*p* = 0.65) and unknown pathogen (*p* = 0.99) (Additional file 1: Table S4). MET with septic shock had higher blood lactate than NO-MET at admission (*p* < 0.001) and during the first 12 h (Fig. 1). Bicarbonate was lower (*p* < 0.01). They also received more renal replacement therapy (*p* = 0.02), while they had less chronic renal failure and there was no significant difference in serum creatinine, pH, day 1 urinary output or acute kidney injury occurrence. In MET, there was a linear correlation between blood lactate and serum creatinine (*ρ* = 0.36; *p* < 0.01) in contrast to NO-MET (*ρ* = 0.09; *p* = 0.41) (Additional file 1: Figures S2 and S3). However, lactate was even higher in MET (*p* < 0.001) with normal kidney function (MDRD creatinine clearance > 60 mL/min/1.73 m^2^).

**Table 2:** Subgroup of ICU diabetics with septic shock: main characteristics at ICU admission, during ICU stay and ICU/hospital outcome

	Septic shocks	No metformin	Metformin
N	131	79 (60.3)	52 (39.7)
Age (y)	70 [63–78]	71 [64–78]	66 [61–78]
Men	89 (67.9)	56 (70.9)	33 (63.5)
SAPS II	52 [42–69]	48 [40–68]	57 [46–68]
Usual metformin contraindication	79 (60.3)	56 (70.9)	23 (44.2)*
Chronic respiratory failure	30 (22.9)	21 (26.6)	9 (17.3)
Chronic cardiac failure	27 (20.6)	20 (25.3)	7 (13.5)
Chronic liver disease	26 (19.8)	18 (22.8)	8 (15.4)
Chronic renal failure	19 (14.5)	16 (20.3)	3 (5.8)*
Recent myocardial infarction	1 (0.8)	1 (1.3)	0
pH	7.32 [7.2–7.38]	7.32 [7.23–7.39]	7.26 [7.17–7.38]
PaCO_2_ (mmHg)	34 [27–42]	35 [29–43]	34 [24–42]
HCO_3_ (mmHg)	18.2 [13.3–22.2]	19.7 [14.7–24.1]	15.5 [10.1–19.9]*
Lactate (mmol/L)	2.2 [1.1–5]	1.4 [1–2.8]	4.5 [2.1–8.7]*
INR	1.5 [1.2–2.3]	1.6 [1.3–2.9]	1.4 [1.1–1.9]
Bilirubin (µmol/L)	12 [8–24]	13 [8–26]	10 [8–19]
C-reactive protein (mg/L)	95 [24–224]	98 [30–225]	85 [14–212]
Haemoglobin (g/dL)	10.6 [9.1–12.4]	10.7 [9.3–12.4]	10.5 [9.1–12.4]
Leucocytes (G/L)	12.1 [8.3–19.6]	11.9 [8.5–19]	12.9 [8.4–21.6]
Platelets (G/L)	185 [119–265]	199 [120–273]	173 [118–252]
Creatinine (µmol/L)	167 [113–326]	163 [108–276]	176 [123–364]
Urinary output day 1 (mL)	1200 [553–2200]	1200 [558–1925]	1425 [443–2400]
Number of patients with vascular filling > 50 mL/kg ≥ 1 day	76 (60.3)	42 (58.4)	34 (69.4)
Maximum dose of noradrenaline			
(mg/h)	2 [1–4.3]	2 [1–3.5]	3.5 [1.3–5]*
(µg/kg/min)	0.43 [0.22–0.95]	0.4 [0.21–0.76]	0.61 [0.23–1.16]
Maximum dose of adrenaline			
(mg/h)	2.5 [1.5–6]	3 [1.5–6.3]	2.5 [1.4–6]
(µg/kg/min)	0.61 [0.25–1.22]	0.52 [0.22–1.3]	0.66 [0.27–0.98]
Noradrenaline duration (h)	39 [18–64]	48 [19–71]	36 [15–59]
Adrenaline duration (h)	36 [9–90]	36 [14–90]	30 [6–102]
Vasopressor duration (h)	48 [24–96]	48 [24–97]	36 [23–72]
Acute kidney injury	104 (79.4)	62 (78.5)	42 (80.8)
ARDS	48 (36.6)	27 (34.2)	21 (40.4)
Renal replacement therapy	51 (38.9)	24 (30.4)	27 (51.9)*
Invasive ventilation	96 (73.3)	56 (70.9)	40 (76.9)
ICU length of stay (d)	9 [5–16]	9 [6–19]	7 [4–13]
Hospital length of stay (d)	15 [7–29]	15 [8–29]	16 [4–26]
ICU death	51 (38.9)	31 (39.2)	20 (38.5)
Hospital death	53 (40.5)	33 (41.8)	20 (38.5)

Values are *n* (%) or median [IQR 25th–75th]

* *p* < 0.05 between metformin and no metformin

**Fig. 2:**
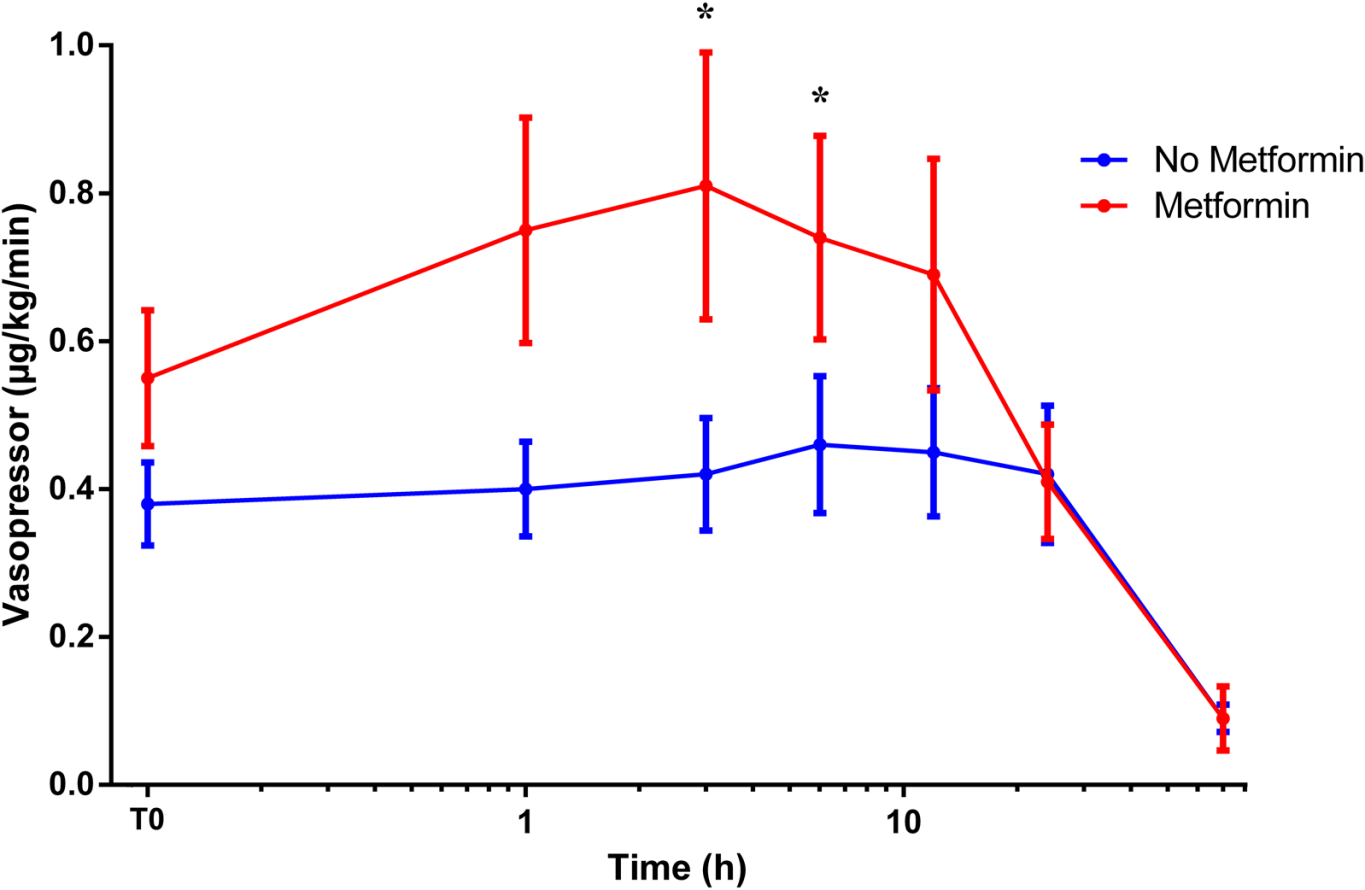
Initial evolution of vasopressor dosage in ICU diabetics sustaining septic shock with or without pre-admission metformin treatment. T0: time of septic shock diagnosis. Abscissa axis is log 10 scale. *p < 0.05

Septic shock severity can also be evaluated by the amount of vascular filling and the dose of vasopressors. There was no difference in the number of patients with intensive vascular filling (i.e. more than 50 mL/kg/day) between MET and NO-MET, but there was a statistical trend for higher maximal dose of noradrenaline in MET (*p* = 0.09). Vasopressor dose was significantly higher in MET the first hours after reaching criteria for septic shock (Fig. 2).

### Mortality and length of stay

ICU or hospital lengths of stay as well as ICU death showed no statistically significant difference between MET and NO-MET in the cohort of diabetics and in the subgroup of septic shock patients. Hospital death was not significantly different in multivariate regression model analysis (OR 0.75 [0.44–1.28]; *p* = 0.29) (Additional file 1: Table S5). In the subgroup of septic shock patients, metformin was associated with a lower mortality after multivariate analysis with odds ratio 0.61 [95% CI 0.36–0.99]; *p* = 0.049 (Table 3).

**Table 3:** Hospital death among septic shock patients: univariate analysis and conditional forward stepwise multivariate analysis with metformin as analysis factor

	Survivor	Non-survivor	*P*-univariate	Odds ratio	*P*-multivariate
*N*	80 (61.1)	51 (38.9)	–	–	–
Men	49 (61.3)	40 (78.4)	0.055	NS	NS
SAPS II	49 [40–61]	65 [46–80]	0.001	1.05 (1.04–1.07)	< 0.001
Metformin (*n*)	32 (40)	20 (39.2)	1	0.61 (0.37–0.99)	0.049
Lactate (mmol/L)	1.7 [1–3.9]	3.2 [1.4–7.1]	0.003	1.21 (1.1–1.34)	< 0.001
ARDS (*n*)	23 (28.8)	25 (49)	0.03	NS	NS
RRT (*n*)	24 (30)	27 (52.9)	0.011	NS	NS
Invasive ventilation (*n*)	48 (60)	48 (94.1)	< 0.001	NS	NS
Urinary output day 1 (mL)	1400 [675–2400]	1030 [65–1900]	0.03	NS	NS

*α* = 0.05. Area under the curve of the multivariate model = 0.786

*RRT* renal replacement therapy, *NS* not significant

Blood lactate levels showed a prognostic value in MET (AUC 67.3% (95% CI 58.3–76.4); *p* = 0.001) and NO-MET (AUC 68.6% (61.5–75.8); *p* < 0.001) of the cohort and also in MET (AUC 66.7% (51.5–81.9); *p* = 0.05) and NO-MET (AUC 65.5% (53–78.1); *p* = 0.02) of shocked patients. But prognostic cut-off value for lactate with the highest sensitivity and specificity was higher in MET (2.15 mmol/L, sensitivity 65%, specificity 61.6%) than in NO-MET (1.35, sensitivity 66.2%, specificity 61.3%). Likewise in the subgroup of septic shock patients, cut-off values were 4.45 mmol/L (sensitivity 57.9%, specificity 56.7%) versus 1.45 mmol/L (sensitivity 58.1%, specificity 56.2%), respectively.

Among MET, there was no significant difference in hospital death between patients with or without usual contraindication (OR 1.24 [0.48–3.2]; *p* = 0.66) (Additional file 1: Table S6).

## Discussion

In our large cohort on critically ill diabetic patients, metformin use before admission to ICU did not affect in-hospital mortality; however, pre-admission metformin treatment was independently associated with a decrease in hospital mortality in the group of septic shock patients, even with an initial clinical presentation appearing more severe. Indeed, independent of kidney function, vasopressor dosages and serum lactate levels were higher during the first hours after shock onset in MET. Nevertheless, metformin did not seem to induce shock per se because there was no more septic shock from unknown aetiology or unknown pathogens in MET than in NO-MET.

A beneficial association between metformin and mortality has been already described both in selected patients with chronic heart failure [11], liver disease [12, 13], mild-to-moderate kidney failure [14] which are usual contraindications, and in ICU patients [3]. In this latter study, based on retrospective analysis of Northern Denmark database, 30-day mortality was lower in metformin users than in non-metformin users with adjusted hazard ratio = 0.8 (95% confidence interval 0.71–0.95). Propensity-score-matched analyses yielded the same results. In our work, more than 90% were medical admissions, whereas two-thirds of the 7404 ICU patients with type 2 diabetes in Christiansen et al.’s study were surgical admissions. However, no data were available concerning septic shocks, vasopressor dosages or even blood lactate levels. Mechanisms of this beneficial effect remain unclear: in ICU patients, metformin may supply higher amounts of lactate serving as an energetic carbon source and therefore is available for ischaemic tissues with glucose preservation. Metformin may also decrease cellular hypoxia of less perfused tissues by decreasing oxygen consumption.

However, clinical severity seems higher in MET. Lactate levels are significantly higher in ICU diabetics with or without septic shock (Additional file 1: Figure S4). This issue still remains controversial with studies finding no effect of metformin on lactate rate [5, 15, 16] or, on the contrary, finding an increased lactate [17–27]. One reason for this discrepancy may be that ICU patients, unlike other patients, suffer acute stress with endogenous catecholamine release leading to increased lactate levels through adrenergic receptor stimulation. Physiological studies showed that metformin enhances lactate production and decreases oxygen consumption [23–25] by inhibiting mitochondrial chain complexes [19, 22–24, 27]. Therefore, in our study, prognostic cut-off values are higher in MET, especially when there is a septic shock, as previously found [28]. It is usually admitted that lactic acidosis in metformin users is due to a reduced renal drug clearance. Lactate and creatinine levels (and creatinine clearance) are linearly correlated in our study as previously shown [17, 18, 21, 26, 29–31]. But lactate levels remain higher in patients without kidney injury with metformin than without. This last issue was only previously described in case reports and one cohort study [26], although another study failed to find hyperlactatemia when kidney function was normal [29]. MET probably received more haemodialysis for the purpose of either correcting deeper hypobasemia or eliminating plasma metformin.

- Vasopressor dosages are higher in septic shock diabetics with pre-admission metformin. This increase in catecholamines need, which has not been previously described, is not due to acidosis per se because pH values are similar with or without metformin. Recent data suggest that metformin decreases adenylate cyclase activity and therefore cyclic AMP concentration [32]. The effects of vasopressors are mediated by adrenergic receptors, G protein and adenylate cyclase stimulations leading to an increase in cyclic AMP concentration. It is assumed that it is necessary to increase vasopressor dosages in order to obtain the same haemodynamic effect and compensate decreased adenylate cyclase activity induced by metformin. Indeed, metformin does not seem to produce sepsis-like shock because there is as much septic shock of unknown aetiology or germ in MET than in NO-MET. However, metformin actually seems to worsen the criteria usually used to assess the severity of septic shocks.

Finally, in our study, patients treated with metformin despite the presence of the usual contraindications do not have higher lactate levels. The mortality rate is not increased either. These contraindications have been challenged for several years so that metformin seems deleterious only in terminal kidney disease [33]. Our collected data did not allow us to evaluate outcome according to the intensity of each organ failure. It is possible that our patients had mainly mild-to-moderate lung, liver, heart or kidney injury that would be insufficient to worsen outcome or lactate level.

Our study is subject to certain limitations. First, it is a retrospective study, avoiding observation bias, but with selection bias due to non-inclusion of patients with missing data. Thus, we cannot determine whether metformin users are more likely to be admitted to ICU than other antidiabetics’ takers, and also whether the presence of a contraindication for its use is linked to a higher rate of hospitalization. The lack of randomization of metformin therapy does not indicate whether the improvement in observed survival is due to metformin itself or whether the clinical presentation and biological characteristics of patients taking metformin appear to be ‘falsely’ more severe. We have included in our logistic regression model certain parameters such as lactate and bicarbonate levels, which are both influenced by the presence of metformin and most likely do not have the same prognostic value in patients previously untreated by metformin. Similarly, elevated doses of vasopressors, which are used as a criterion for poor outcome for example in the SOFA score, may not carry the same prognostic significance. Metformin blood dosage has never been performed. However, it seems linearly correlated to lactate concentration [18, 21, 31]. Lastly, comparison between MET treated or non-treated by renal replacement therapy was unfeasible because analysis would lack power and be statistically unreliable. If current scientific opinion suggests its use in metformin overdose, there is no strong proof. There is indeed a contradiction between studies finding a beneficial association between sepsis and metformin and in contrast the desire to eliminate metformin by haemodialysis. Therefore, we suggest that future studies should seek to answer two questions: Is there a benefit in giving metformin during the first hours of septic shock in diabetic patients previously untreated by metformin? Is there really a benefit in the early elimination of metformin by haemodialysis in diabetic patients with septic shock and without acute kidney injury?

## Conclusions

Metformin use before admission to ICU is associated with a decrease in mortality in septic shock patients despite a worse clinical presentation on admission. Metformin users have higher lactate levels independent of kidney function and need higher vasopressor dosages during the first hours of septic shock. Metformin does not seem to induce shock per se. The presence or absence of one of the usual contraindications to taking metformin does not alter lactate levels or hospital mortality.

## Additional file

**Additional file 1: Figure S1.** Study flowchart. **Figure S2.** Linear regression between blood creatinine and lactate levels in metformin users patients. **Figure S3.** Linear regression between blood creatinine and lactate levels in non-metformin users patients. **Figure S4.** Lactate levels. **Table S1.** Main admission pattern of ICU-admitted diabetics. **Table S2.** ICU-admitted diabetics with preadmission metformin treatment with or without usual metformin contraindication. **Table S3.** Aetiologies and germs responsible for septic shocks in ICU-diabetics. **Table S4.** Septic shocks without aetiology at the end of hospital stay. **Table S5.** Hospital death among ICU-admitted diabetic patients: univariate analysis and conditional forward stepwise multivariate analysis with metformin as analysis factor. **Table S6.** Hospital death among metformin patients: univariate analysis and conditional forward stepwise multivariate analysis with usual contraindication as analysis factor.

## Abbreviations

AMP: adenosine monophosphate
ARDS: acute respiratory distress syndrome
AUC: area under the curve
ICU: intensive care unit
KDIGO: Kidney Disease Improving Global Outcome
MALA: metformin-associated lactic acidosis
MDRD: Modification of Diet in Renal Disease
MET: metformin users
NO-MET: non-metformin users
OAD: oral antidiabetic
ROC: receiver operating characteristic
RRT: renal replacement therapy
SAPS II: Simplified Acute Physiology Score II
SOFA: Sepsis-Related Organ Failure Assessment score
