# Supplementary material for: Does metformin exposure before ICU stay have any impact on patients’ outcome? A retrospective cohort study of diabetic patients
Source: Ann Intensive Care. 2017 Dec 2;7:116. doi: 10.1186/s13613-017-0336-8 (PMC5712297; doi:10.1186/s13613-017-0336-8)
Supplement: Supplementary file 1 — Additional file 1: Figure S1. Study flowchart. Figure S2. Linear regression between blood creatinine and lactate levels in metformin users patients. Figure S3. Linear regression between blood creatinine and lactate levels in non-metformin users patients. Figure S4. Lactate levels. Table S1. Main admission pattern of ICU-admitted diabetics. Table S2. ICU-admitted diabetics with preadmission metformin treatment with or without usual metformin contraindication. Table S3. Aetiologies and germs responsible for septic shocks in ICU-diabetics. Table S4. Septic shocks without aetiology at the end of hospital stay. Table S5. Hospital death among ICU-admitted diabetic patients: univariate analysis and conditional forward stepwise multivariate analysis with metformin as analysis factor. Table S6. Hospital death among metformin patients: univariate analysis and conditional forward stepwise multivariate analysis with usual contraindication as analysis factor. [file 13613_2017_336_MOESM1_ESM.doc]

**ELECTRONICAL SUPPLEMENTAL MATERIAL (ESM)**

***Does Metformin exposure before ICU stay have any impact on patients’ outcome?***

Sebastien JOCHMANS, MD (1,2) ; Jean-Emmanuel ALPHONSINE, MD (3) ; Jonathan CHELLY, MD (1,2) ; Ly Van Phach VONG, MD (1) ; Oumar SY, MD (1) ; Olivier ELLRODT, MD (1) ; Nathalie ROLIN, MD (1) ; Mehran MONCHI, MD (1,2) ; Christophe VINSONNEAU, MD (4)

**Authors Affiliation:**

1 Intensive Care Medicine Department, Melun Hospital, Melun ZIP 77000, France

2 Clinical Research Unit, Melun Hospital, Melun ZIP 77000, France

3 Medical Intensive Care Unit, AP-HP Bicêtre Hospital, Le Kremlin-Bicêtre ZIP 94270, France

4 Mixed Intensive Care Unit, Bethune Hospital, Bethune ZIP 62408, France

**ESM Content: 4 Figures (Figure S1 to Figure S4) and 6 tables (Table S1 to Table S6).**

**Figure S1. Study flowchart**


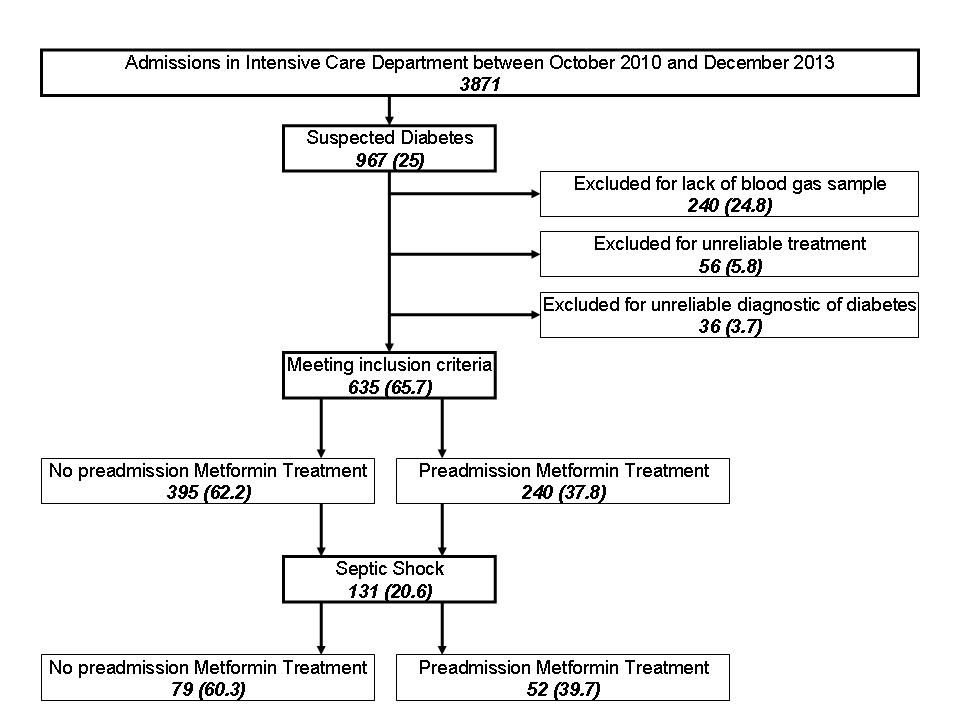


**Figure S2. Linear regression between blood creatinine and lactate levels in metformin users patients.** Axis scales are Log10; continuous line is regression line; dotted line is 95% confidence interval. R^2^ = 13.2%; p = 0.009

**Figure S3. Linear regression between blood creatinine and lactate levels in non-metformin users patients.** Axis scales are Log10; continuous line is regression line; dotted line is 95% confidence interval. R^2^ = 0.88%; p = 0.41

**Figure S4. Lactate levels.** * p < 0.05; ** p < 0.001.


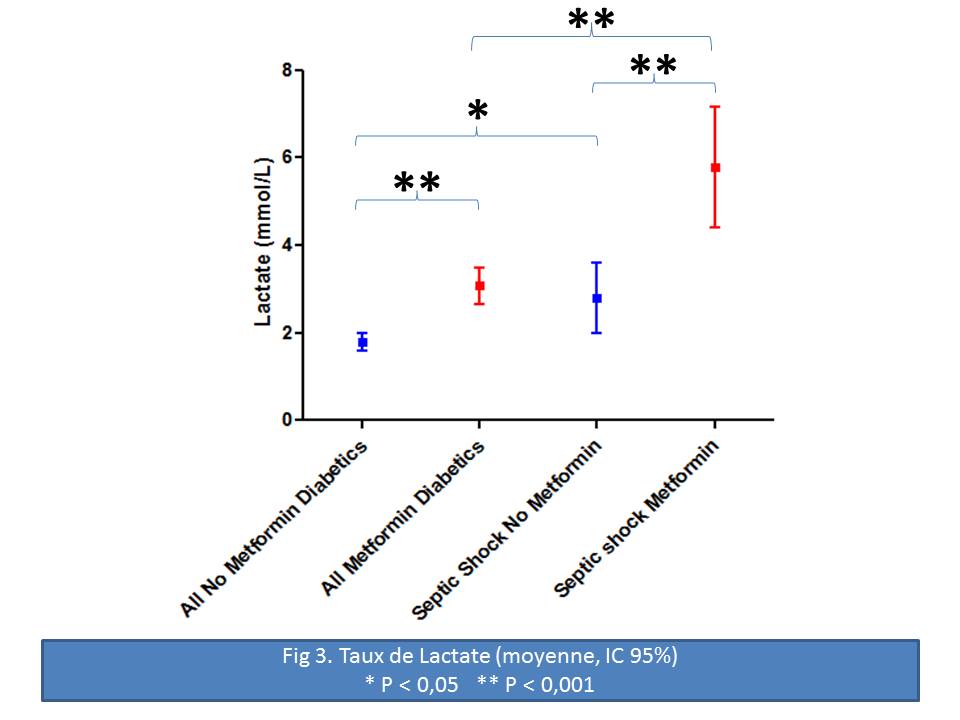


**Table S1. Main admission pattern of ICU-admitted diabetics.** No statistical difference between groups with α = 0.05.

|  | **ICU-Diabetics** | **No Metformin** | **Metformin** |
| --- | --- | --- | --- |
| **Medical admission** | 588 (92.6) | 367 (92.9) | 221 (92.5) |
| Acute respiratory failure | 266 (41.9) | 175 (44.3) | 91 (37.9) |
| Septic shock | 65 (10.2) | 40 (10.1) | 25 (10.4) |
| Other shock | 33 (5.2) | 15 (3.8) | 18 (7.5) |
| Acute kidney injury | 63 (9.9) | 42 (10.6) | 21 (8.8) |
| Coma | 48 (7.6) | 25 (6.3) | 23 (9.6) |
| Acute metabolic complication of Diabetes | 36 (5.7) | 27 (6.8) | 9 (3.8) |
| Other medical | 65 (10.2) | 37 (9.4) | 28 (11.7) |
| **Surgical admission** | 47 (7.4) | 28 (7.1) | 19 (7.5) |
| Abdominal surgery | 28 (4.4) | 16 (4.1) | 12 (5) |
| Other surgery | 19 (3) | 12 (3) | 7 (3) |

**Table S2. ICU-admitted diabetics with preadmission metformin treatment with or without usual metformin contraindication.** Values are n (%) or mean ± SD (95% Confidence Interval).

|  | **No Contraindication** | **Contraindication** | **p** |
| --- | --- | --- | --- |
| N | 121 (50.4) | 119 (49.6) | - |
| **Men (n)** | 60 (49.6) | 93 (78.2) | **< 0.001** |
| **Age (y)** | 69 [60 – 79] | 67 [60 – 77] | 0.89 |
| **IGS2** | 40 [29 – 52] | 37 [30 – 50] | 0.98 |
| **pH** | 7.33 ± 0.14 [7.31 – 7.36] | 7.33 ± 0.14 [7.31 – 7.36] | 0.82 |
| **PaCO2 (mmHg)** | 34.6 ± 12.7 [32.3 – 36.9] | 38.8 ± 14.3 [36.2 – 41.4] | **< 0.02** |
| **HCO3 (mmol/L)** | 18.9 ± 7 [17.7 – 20.1] | 20.9 ± 8.3 [19.4 – 22.4] | **< 0.05** |
| **Lactate (mmol/L)** | 3.1 ± 3.18 [2.53 – 3.67] | 3.03 ± 3.44 [2.41 – 3.65] | 0.86 |
| **INR** | 1.52 ± 1 [1.33 – 1.71] | 1.95 ± 1.7 [1.63 – 2.27] | **0.03** |
| **Kidney Failure (n)** | 62 (51.2) | 62 (52.1) | 0.89 |
| **ICU LOS (d)** | 6 [3 – 9] | 6 [4 – 9] | 0.68 |
| **ICU death (n)** | 21 (17.4) | 20 (16.8) | 0.91 |
| **Renal Replacement Therapy (n)** | 19 (15.7) | 22 (18.5) | 0.57 |
| **Vasopressors (n)** | 49 (40.5) | 44 (37) | 0.58 |
| **Invasive ventilation (n)** | 51 (42.1) | 40 (33.6) | 0.17 |

**Table S3. Aetiologies and germs responsible for septic shocks in ICU-diabetics.** ESBL: Extended Spectrum Betalactamase; MR: Methicillin Resistant; MS: Methicillin Sensitive.

* 7 patients had multiple germs identified (in cellulitis, peritonitis, cholangitis and pneumonia)

| ***Aetiologies of Septic Shock, n (%)*** | | 131 (100) |
| --- | --- | --- |
| Pneumonia | | 71 (54.2) |
| Pyelonephritis | | 16 (12.2) |
| Peritonitis | | 9 (6.9) |
| Cellulite | | 7 (5.3) |
| Others < 5% | | 24 (18.3) |
| ***Unknown Aetiology*** | | 4 (3.1) |
| ***Patients without positive bacteriologic sample*** | | 57 (43.5) |
| ***Germs, n (%) of positive bacteriologic sample**** | | - |
|  | Escherichia Coli | 21 (25.6) |
|  | Streptococcus Pneumoniae | 11 (13.4) |
|  | Staphylococcus Aureus MS | 8 (9.8) |
|  | Klebsiella Pneumoniae | 4 (4.9) |
|  | Staphylococcus Aureus MR | 3 (3.7) |
|  | Pseudomonas Aeruginosa | 3 (3.7) |
|  | Bacillus GRAM negative with ESBL | 3 (3.7) |
|  | Virus | 1 (1.2) |
|  | Others < 3% | 28 (34.1) |

**Table S4. Septic shocks without aetiology at the end of hospital stay.** Values à n (%).

|  | **No Metformin** | **Metformin** | **p-value** |
| --- | --- | --- | --- |
| **Aetiology found** | 77 (97.5) | 50 (96.2) | **0.65** |
| **No Aetiology found** | 2 (2.5) | 2 (3.8) |
| **Germ found** | 44 (55.7) | 29 (55.8) | **0.99** |
| **No germ found** | 35 (44.3) | 23 (44.2) |

**Table S5. Hospital death among ICU-admitted diabetic patients: univariate analysis and conditional forward stepwise multivariate analysis with metformin as analysis factor.** RRT: Renal Replacement Therapy; CRP: C-Reactive Protein; NS: Not Significant. α = 0.05. Area Under Curve of the multivariate model = 0.829

| **All Diabetics** | **Survivor** | **Non Survivor** | **P-Univariate** | **Odds Ratio** | **P-Multivariate** |
| --- | --- | --- | --- | --- | --- |
| **N** | 520 (81.3) | 115 (18.7) | - | - | - |
| **Age (y)** | 70 [60 – 78] | 75 [66 – 82] | < 0.001 | 1.05 (1.03-1.08) | < 0.001 |
| **Men (n)** | 329 (63.5) | 79 (68.7) | 0.27 | NS | NS |
| **SAPS 2** | 37 [30 – 47] | 56 [44 – 74] | < 0.001 | 1.04 (1.02-1.05) | < 0.001 |
| **Metformin (n)** | 199 (38.3) | 40 (34.8) | 0.48 | 0.75 (0.44-1.28) | 0.29 |
| **Usual Metformin contraindication (n)** | 313 (60.4) | 74 (63.3) | 0.6 | NS | NS |
| **pH** | 7.37 [7.29 – 7.43] | 7.33 [7.22 – 7.39] | < 0.001 |  |  |
| **PaCO2 (mmHg)** | 36 [29 – 43] | 36 [29 – 51] | 0.37 | NS | NS |
| **HCO3 (mmol/L)** | 21.9 [17.1 – 25.4] | 19.7 [15.2 – 23.8] | 0.008 |  |  |
| **Lactate (mmol/L)** | 1.3 [0.8 – 2.2] | 2.1 [1.3 – 4.3] | < 0.001 | 1.13 (1.03-1.24) | 0.007 |
| **INR** | 1.2 [1 – 1.6] | 1.6 [1.2 – 2.3] | < 0.001 | NS | NS |
| **Bilirubin (µmol/L)** | 10 [7 – 15] | 12 [8 – 22] | < 0.001 | 1.02 (1.01-1.03) | 0.002 |
| **CRP (mg/L)** | 32 [8 – 113] | 53 [ 11 – 133] | 0.07 | NS | NS |
| **Haemoglobin (g/dL)** | 11.3 [9.8 – 13] | 10.8 [9.2 – 12.7] | 0.08 | NS | NS |
| **Leucocytes (G/L)** | 10.8 [7.8 – 14.4] | 13 [8.6 – 17.6] | 0.002 | NS | NS |
| **Platelets (G/L)** | 217 [159 – 278] | 199 [135 – 274] | 0.14 | NS | NS |
| **Creatinine (µmol/L)** | 130 [82 – 233] | 139 [96 – 246] | 0.08 | NS | NS |
| **Acute kidney injury (n)** | 308 (59.2) | 85 (73.9) | 0.005 | NS | NS |
| **ICU LOS (d)** | 6 [4 – 9] | 6 [3 – 14] | 0.02 | - | - |
| **Hospital LOS (d)** | 14 [8 – 24] | 7 [3 – 15] | < 0.001 | - | - |
| **RRT (n)** | 77 (14.8) | 36 (31.3) | < 0.001 | NS | NS |
| **Vasopressors (n)** | 144 (27.7) | 85 (73.9) | < 0.001 | 2.67 (1.59-4.49) | < 0.001 |
| **Invasive ventilation (n)** | 137 (26.3) | 93 (80.9) | < 0.001 | 3.97 (2.11-7.46) | < 0.001 |

**Table S6. Hospital death among metformin patients: univariate analysis and conditional forward stepwise multivariate analysis with usual contraindication as analysis factor.** RRT: Renal Replacement Therapy. α = 0.05. Area Under Curve of the multivariate model = 0.778

| **Parameters** | **Survivors** | **Non-survivors** | **P-Univariate** | **Odds Ratio** | **P-Multivariate** |
| --- | --- | --- | --- | --- | --- |
| **Age (y)** | 66 [58-77] | 73 [65-79] | 0.003 | 1.04 (1-1.09) | 0.07 |
| **Hb (g/dL)** | 12 [10.3-13.5] | 10.1 [8.6-11.9] | < 0.001 | 0.84 (0.7-1) | 0.06 |
| **Creatinine (µmol/L)** | 106 [76-176] | 118 [92-168] | 0.020 | 1 (1-1.01) | 0.22 |
| **Invasive ventilation (n)** | 53 (27.6) | 38 (79.2) | < 0.001 | 4.5 (1.4-14.5) | 0.01 |
| **RRT (n)** | 24 (12.5) | 17 (35.4) | < 0.001 | 2.4 (0.51-11.2) | 0.27 |
| **Vasopressor (n)** | 57 (29.7) | 36 (75) | < 0.001 | 2.48 (0.86-7.11) | 0.09 |
| **Lactate (mg/L)** | 1.6 [1-3.2] | 3.4 [1.5-7] | < 0.001 | 1.21 (1.01-1.43) | 0.03 |
| **SAPS 2** | 27 [36-46] | 54 [42-75] | < 0.001 | 1 (1-1.05) | 0.08 |
| **pH** | 7.36 [7.28-7.42] | 7.35 [7.21-7.39] | 0.005 | 231 (0.25-2.10^6^) | 0.12 |
| **Usual contraindication (n)** | 93 (48.4) | 26 (54.2) | 0.767 | 1.24 (0.48-3.2) | 0.66 |
